# Comment on Balàka et al. Updated Checklist of Chondrichthyan Species in Croatia (Central Mediterranean Sea). *Biology* 2023, *12*, 952

**DOI:** 10.3390/biology13030135

**Published:** 2024-02-21

**Authors:** Alen Soldo, Lovrenc Lipej

**Affiliations:** 1 Department of Marine Studies, University of Split, 21000 Split, Croatia; 2 Marine Biology Station, National Institute of Biology, 6330 Piran, Slovenia; lovrenc.lipej@nib.si

The authors write that the last checklist for Croatia was carried out in 2009 and that 52 chondrichthyan species were reported in Croatian waters [1] based on a report from Serena et al. [2]. However, that is inaccurate as considering the area of Croatian waters within the Adriatic and the length of its coast, all the checklists made so far, including general fish lists, by the Croatian and regional authors, always referred to the whole Adriatic Sea, not to the portion of Croatian waters. Although the national checklist might be useful in the case of countries occupying small Adriatic areas, in the case of Croatia, which has a coastline more than three times longer than all other Adriatic countries combined (the total length of Croatia’s coast is 6278 km, of which 1880 km belongs to the mainland and 4398 km to the coastline of 1244 islands [3]), this has not been considered useful so far. With regard to the mentioned reference [2], as its coauthors, we can say that the main purpose of that checklist was to be used during the preparation of the Action Plan for the Conservation of Cartilaginous (Chondrichthyan) Fishes in the Mediterranean on a national level. Moreover, the checklist was never peer-reviewed, and thus it was not considered the official checklist, as is further supported by Soldo and Lipej [4], who never mentioned that reference. The only official checklists of chondrichthyans in the Adriatic Sea, and thus also in Croatia, apart from the general checklist of the Adriatic fishes, are from 1999 [5] and the recent one from 2022 [4].

In the paper, the authors raise doubts about the correct identification of *Carcharias taurus* Rafinesque, 1810 caught in 1999 off the island of Molat, as initially published by Lipej et al. [6]. The authors obtained a new photo (Figure 7) similar to the initially published Figure 10 in Lipej et al. [6]. Based on the characteristics visible in these photos, the authors concluded that the specimen is *Odontaspis ferox* (Risso, 1810) and not *C. taurus*. To support such an opinion, the authors are referring to the coloration of the specimen, although it is known that coloration cannot be used to distinguish these similar species as various color patterns, including the same for different species, have been observed so far [7] and explained as a phenotypic variation [8]. Additionally, the authors, in their reply to this Comment, again referred to spot patterns associated with *C. taurus* and considered that a lack of these spots is unlikely for this species and represents a rare case; thus, they “are confident that colouration can provide supportive evidence for correct species identification”. However, Castro [9] clearly states that the spots in *C. taurus* fade with age and disappear by the time the animals reach 180–200 cm, which is undoubtedly smaller than the size of the specimen in the case. The authors are also referring to the position of the dorsal fin and its distance from the pectoral and pelvic fin, which also cannot be used for a positive identification. Firstly, the dorsal fin position is a known variation [10], and secondly, the claim of an accurately measured distance from the available photos, in which the whole dorsal fin is not clearly visible, is very arbitrary. Furthermore, the authors express their opinion that the eyes of the specimen are large, while small in *C. taurus*, which is also an arbitrary inference, as according to Compagno [8], the eyes of *C. taurus* are about 0.9 to 1.4% of the total length, while in *O. ferox*, they are about 1.6 to 2.8% of the total length. Those differences are so small that it is impossible to verify them from the available photos. In their reply, the authors stated that the eyes are approximately 1.8% of the total length but also considered these measures only as approximate values, as no exact measurements could be taken. Therefore, we do not have any further comments on this issue. On the other hand, the authors have not used other distinctive characteristics that point to *C. taurus*, e.g., both Compagno [8] and Castro [9] state that *C. taurus* has a first dorsal fin about as large or slightly larger than the second dorsal fin and anal fin, while in *O. ferox* the first dorsal fin is noticeably larger (about double in size) than the second dorsal fin and anal fin. Examining those characteristics on available photos points to *C. taurus*, as the size of both dorsal fins seems the same.

However, what authors have not considered is the fact that Lipej et al. [6] did not base their final identification of the species on the previously mentioned characteristics seen in the photo, because they were reluctant to use them as proof, particularly because not all the characteristics were clearly visible. Thus, the positive identification of the specimen in the case was made according to another available photo published as Figure 11 in Lipej et al. [6], which shows details of teeth and jaws that undoubtedly correspond with *C. taurus*. Jaw and dental characteristics are the main distinctive characteristics used for the distinction between these species, as other body characteristics may vary [4,6]. The authors, in their reply, discuss the rare variation in the number of lateral cusplets between *O. ferox* and *O. noronhai*, but that variation is not relevant to *C. taurus*. Thus, we suggest that any identification of these species based solely on uncertain body photos should be avoided if jaw and dental characteristics are not available.

Another issue is the inclusion of highly doubtful species in a list of Croatian chondrichthyans, and thus also the Adriatic ones. The first issue is related to *Scyliorhinus duhamelii* (Garman, 1913), which is included in the list due to records “recently collected by citizen scientists in northern Croatia”. The presence of this species in the Adriatic is based solely on the report of Soares and Carvalho [11], who examined several specimens from the Adriatic Sea and concluded that this species can be distinguished from *Scyliorhinus canicula* (Linnaeus, 1758) by its color pattern, internasal distance, shape of anterior nasal flaps and clasper components. It has to be noted that none of the published fish checklists, including those published after Soares and Carcalho [11], has ever included this species as the Adriatic one. Hence, Serena et al. [12] disagreed with the opinion of Soares and Carvalho [11] and considered this species to be invalid for the Mediterranean Sea and suggested that to clarify this issue more detailed studies, including molecular taxonomy analysis, should be undertaken. That being said, a study [13] that tested the population genetic structure among populations of *S. canicula* collected across European seas, including the Adriatic, found only *S. canicula* as the valid species. The study showed a congruence in patterns of population structure identified between the mtDNA and microsatellite data across the European seas but also found significant genetic differences among populations across the Mediterranean basin, especially in the eastern part, which might be an explanation for the hypothesis of Soares and Carcalho [11]. Therefore, without more solid data, *S. duhamelii* cannot be considered the Adriatic species.

A similar case involves the inclusion of *Squatina aculeata* Cuvier, 1829, which was never reported as the Adriatic species by any of the lists. The authors explain that its inclusion was by examination of the 26 cm long juvenile apparently collected in Split around the year 1900 and deposited in the Natural History Museum Vienna, Austria. Although the authors state that historical data pose significant problems in identification due to poor preservation, such as fading colors and changes in the shape of the body, e.g., skin wrinkles, and that distinguishing between *Squatina squatina* (Linnaeus, 1758), which is the Adriatic species, and *S. aculeata* based on morphology alone has always been difficult, especially in juveniles, they decided to use that old single record as a reason for declaring *S. aculeata* as the Adriatic species. According to our opinion, any inclusion of a new species needs more validated evidence. The authors, in their reply, mentioned that an additional record of *S. aculeata* was reported by Holcer and Lazar [14] but it has to be noted that the record refers to the specimen from 1939 initially reported as *Squatina fimbriata*, which is considered solely as a questionable and ambiguous synonym of *S. aculeata* [15], and it thus remains unknown which species was originally reported.

The authors’ opinion on *Sphyrna tudes* (Valenciennes, 1822), which is excluded from most of the Adriatic lists, is a bit confusing as although it is listed by the authors in Table 1, later, it is written that this species is excluded from the list. 

Other issues are related to the last records of some species which may inaccurately describe their status in the Adriatic; e.g., the authors report the last record of *Chimaera monstrosa* Linnaeus, 1758, on 24th June 1996, while Isajlović [16] reports that in deep waters of the Adriatic this chimaera is considered one of the most abundant species, as it was recorded at 36,4 % of all stations. Isajlović [16] also reports records of *Raja radula* Delaroche, 1809, while authors state that the last record of that species originates from 1984. Also, the status of *Raja asterias* Delaroche, 1809, is confusing as although this species is considered common for the Adriatic, authors have reported a last record from the period 1994–1998. On the other hand, recently, a paper was published by Sviben et al. [17], based on the bachelor’s thesis performed by a student under the tuition of L. Lipej, which reported data on the diet of *R. asterias* based on an examination of 88 specimens collected in Slovenian, Italian and Croatian waters of the northern Adriatic Sea. 

However, statuses are minor issues and not the primary reason for this comment. To conclude: authors are encouraged that in the absence of verified evidence, the identification of species and subsequent publishing of such arbitrary observations should be evaded in order to avoid further confusion related to the status of chondrichthyans, which is a constant problem, particularly in the Mediterranean.
